# An overview of CBDCs and their potential role in the green economy

**DOI:** 10.12688/openreseurope.19970.1

**Published:** 2025-04-24

**Authors:** Christos Kontzinos, Maria Flouri, Paanagiotis Kokkinakos, Konstantinos Alexakis, Fotis Siouzos, Vangelis Marinakis

**Affiliations:** 1 Decision Support Systems Laboratory, School of Electrical & Computer Engineering, National Technical University of Athens, Iroon Polytechniou 9, Zografou, 15780, Greece

**Keywords:** CBDC, Review, Advantages, Disadvantages, Blockchain, Green Economy

## Abstract

In recent years, there has been an ever-intensifying discussion around the use and establishment of Central Bank Digital Currencies (CBDC) in the global economy. This paper examines the reasons why central banks are aiming to introduce CBDCs into the economy, as well as the ways in which the use of CBDCs could contribute to the transition to the Green Economy, focused mainly around the area of providing financial means and incentives towards green investments, green renovations, and more sustainable energy consumption practices. Aiming to provide an all-around and concise overview of CBDCs, this paper explores their technological background, as well as the areas in which they will mainly contribute, as a means of transaction or value storage. Special mention is also made of the initiatives undertaken by the European Central Bank for the issuance of the digital euro as well as the legal and technological framework within which it could operate, to serve the objectives of the EU. Finally, the potential role of CBDCs in the green economy is examined, and ways in which they could be used as a means of supporting individuals and businesses investing in this direction are presented. This publication is written in the context of the Horizon Europe funded project FORTESIE.

## Plain Language Summary

This paper presents an overview of Central Bank Digital Currencies (CBDCs), which are digital versions of national currencies issued and regulated by central banks. Their potential to improve payment systems—by making transactions faster, cheaper, and more secure—has been widely discussed in recent years. In this study, particular attention was given to how CBDCs could contribute to the green economy, which focuses on environmentally friendly investments and practices. The paper examines how such digital currencies could be used to support projects related to energy efficiency, renewable energy, and sustainable business models. Possibilities such as facilitating green investments and offering financial inclusion to individuals without access to traditional banking systems were explored. The technological foundations of CBDCs, including blockchain and smart contracts, were also discussed. In addition, initiatives related to the potential issuance of a digital euro within the European Union were reviewed, along with legal and privacy considerations that would need to be addressed for such systems to function effectively. Although the use of CBDCs in environmental policy remains at an early stage, it was concluded that they hold significant value for enhancing financial systems and supporting sustainability goals. By enabling more direct and transparent financial flows, CBDCs could serve as a valuable tool in the global transition to a greener and more inclusive economy.

## Introduction

Nowadays, physical forms of currency such as coins, banknotes, and checks are taking a backseat due to the radical increase in use of digital currencies and digital forms of payment ( Auer *et al.*, 2022). Even credit and debit cards are being slowly but steadily replaced by digital wallets, e-banking, and m-banking applications as more and more people are becoming technologically proficient in a global scale ( Choi, 2018). Towards the direction of digitising transactions and developing new forms of digital currencies, new technologies such as stablecoins have made their appearance. Stablecoins are types of digital assets, which have a value comparable to that of conventional currencies such as the dollar or the euro and made their appearance as blockchain and cryptocurrencies gained more traction ( Arner *et al.*, 2020).

Central Bank Digital Currencies (CBDCs) are another relatively recent financial innovation that has drawn major scientific interest ( Ozili, 2022b). Despite that fact, the research bibliography lacks a uniform and universally accepted definition of the term, other than the well-accepted fact that they consist of a digital representation of a physical currency that is issued by a central bank. According to a report from the Committee on Payments and Market Infrastructures (CPMI), CBDCs can be defined as “a digital form of money, which is issued by the Central Bank, and is different from deposits in reserve or settlement accounts. In other words, it is an obligation of the Central Bank, expressed in an existing accounting unit, which serves both as a medium of exchange and as a means of storing value” ( CPMI, 2018). Further, according to a definition by the International Monetary Fund, CBDC is “a new form of money, digitally issued by the Central Bank and intended to serve as a legal tender” ( Mancini-Griffoli *et al.*, 2018).

This paper’s main objective is to provide an overview of CBDCs, mostly focusing on their potential impact for the EU and the green economy and is written under the context of the Horizon Europe funded project FORTESIE that aims to design, demonstrate, validate and replicate innovative renovation packages in the building industry with Smart Performance-Based guarantees and CBDC-based financing. 

The current paper is structured as follows: chapter 1 provides the introduction and scope of the paper as well as some introductory definitions of the term CBDC. Chapter 2 presents the background of CBDCs, their types, as well as their advantages and disadvantages. Chapter 3 presents the findings of the bibliographic review that was performed in the context of the paper. Chapter 4 describes the technological background of CBDCs that is tightly related to blockchain technology. Chapter 5 describes the potential impact of CBDCs in the form of a “digital euro” for the EU as well as legal and ethical issues that must be tackled and that mostly relate to user privacy. Chapter 6 presents potential applications and practices of CBDCs in the green economy, and chapter 7 concludes the document.

## Background of CBDCs: taxonomy, global developments, advantages, and disadvantages

Central banks can issue two primary forms of CBDCs: retail (or general purpose) and wholesale. Retail CBDCs are designed for use by the general public and are classified based on their structure—either token-based or account-based ( Mersch, 2017). In contrast, wholesale CBDCs, which are intended for interbank transactions, are exclusively token-based. Token-based systems represent digital or physical currency and derive their value from the issuing central bank. These tokens are accessed using cryptographic keys, much like cryptocurrencies, and can support a degree of user anonymity. Αccount-based CBDCs operate through digital accounts managed by the central bank and require user identification for access ( Bech & Garratt, 2017).

Real life applications of CBDC are few but more and more are being developed and piloted by several countries. The Atlantic Council’s CBDC Tracker is a useful tool that visualises the latest news in CBDC (Atlantic Council, CBDC Tracker). According to it, as of the time of writing this paper, 3 countries have officially launched CBDCs, 36 are in the pilot stage, 30 are in development and 44 are in early research stages. This increased interest in the usefulness of CBDCs is apparent by the fact that 19 out of the G20 countries are in advanced stages of CBDC experimentation.

At this point, and given the increase in CBDC experimentation, piloting, and application, it is worth providing a short analysis of their advantages and disadvantages. Many researchers emphasise that CBDCs should facilitate low-cost payments that are secure and easy to do by everyone, regardless of their technological capacity to ensure their adoption and acceptance by both consumers and businesses ( Huynh *et al.*, 2020). Furthermore, they could provide a way to make the banking system of a country more inclusive towards low-income individuals who might not possess a bank account, which means that they cannot use digital banking services. As such, CBDCs could help low-income individuals to have access to these tools with close to zero cost ( Gnan & Masciandaro, 2018). For most people that do have a bank account, CBDCs would promote transactions with higher efficiency and lower cost ( Koumbarakis & Dobrauz-Saldapenna, 2019). Specifically, without the need for intermediaries such as banks, a widespread use of CBDCs could improve settlement speed and enable real-time payments ( Wadsworth, 2018). CBDCs could also provide a means to combat illegal practices, such as corruption and money laundering, due to the inane ability of their underlying technology (i.e., blockchain) to effectively trace transactions and their details in the form of metadata ( PWC, 2019). In addition, a CBDC could stimulate competition in payment systems and pressure private financial institutions to introduce significant innovations in their operations. Furthermore, CBDCs, as a new form of currency would increase competition between commercial banks, which would translate to more favourable terms for consumers ( Koumbarakis & Dobrauz-Saldapenna, 2019). Finally, a country's favourable attitude toward digital currencies could not only lead to a better national economic status but also create spin-offs in other technology sectors, such as application development and transaction security, which would have a positive effect in job creation and economic development among others.

However, there are also downsides to the introduction of CBDCs. Initially, they face a series of geographical restrictions, since they are accepted only in the country that issued them. In addition, it should be emphasised that CBDCs are heavily reliant on the Internet and smartphone technology. This means that the wide adoption of CBDCs might face some issues as there are still many citizens who do not own smartphones or have access to the Internet. In several rural areas all over the world, internet connections and smart device penetration rates are low, which also affects the potential of a country to mass-adopt CBDCs. Furthermore, even technologically savvy people might require some form of training on the security and functionalities of CBDCs, which would require some overhead from the side of the central bank ( Lee *et al.*, 2021). Finally, there are some issues that could further challenge the wide application of CBDCs that mostly relate to the parallel issuing of both physical currency and CBDCs, which could result in central banks losing some of their income ( Olson, 2018) and in an increased effort required from economists and policymakers to ensure that the potential downsides of CBDCs do not lead to a destabilising economy ( Lee *et al.*, 2021).

## Bibliographic review

This section includes a short bibliographic review of CBDC-related scientific publications that aims to showcase the different thematic areas that are being investigated as well as theoretical and practical approaches of CBDC development and application. In order to obtain the data, research articles, studies, and other types of publications, which had been published during the last five years (2017–2022) and related to the issue under study were studied and assessed. Table 1 below, presents the aforementioned paper thematic, the keywords used for each search, and finally the number of articles that each search returned. While most thematic areas, returned a satisfactory number of publications, the same cannot be said for CBDC applications in the Green Economy which seem to be lagging, most likely due to the immaturity of the technology and its applications, which makes it harder to apply it in domains other than the banking and financial sectors.

**Table 1.** Results of CBDC Bibliographic Review.

Paper Thematic	Keywords	Number of Articles
Classification, Functional Architecture of CBDCs	Central Bank Digital Currency (CBDC), function, architecture, taxonomy, classification	251
CBDC Advantages and disadvantages	Central Bank Digital Currency (CBDC), advantages/ benefits, drawbacks/ disadvantages	158
CBDCs Technology Background	Central Bank Digital Currency (CBDC), technological background, DLT, blockchain	146
Applications in the Financial Sector & Easy Payments	Central Bank Digital Currency (CBDC), application, financial sector, easy payments	173
Perspectives of CBDCs in the European Economy	CBDCs, perspectives, potential, European economy, digital euro, Eurozone	176
Digital Euro & Legal Issues	Digital Euro and legal considerations, legal framework	71
CBDC Implications on the Banking System	CBDC, banks, bank intermediation impact, banking sector	189
CBDC Applications in a Green Economy	CBDC, applications, green economy, circular economy, green finance, sustainable development	14

The Scopus electronic database was used for the search of the data, in which searches were carried out with keywords related to the individual characteristics of the CBDCs studied: i.e. architecture, classification, operation, disadvantages/advantages, their technological background, their uses in the financial sector and easy payments. Regarding the technological background of CBDCs, most approaches in the bibliography use Distributed Ledger Technologies (DLTs) and blockchain to develop CBDC architectures and applications. As such, we added these two terms in the keywords used to research technological papers about CBDCs. In addition, research was conducted regarding the prospects of CBDCs in the European economy, the legal ramifications of the creation of a digital euro and its effects on the European banking system. Finally, for the data search regarding the applications of CBDCs in the green economy, a series of terms related to it such as circular economy, green finance and sustainable development were used as keywords in addition to the term green economy.

## Technological framework of CBDCs

A careful review of the research bibliography shows that the main technology that is being explored and investigated for CBDC development is DLT, the most common expression of which is the blockchain ( Auer & Böhme, 2020). It should be noted that blockchain’s native properties for security, decentralisation, anonymity, and transparency are integral for CBDC system development, similar to the development of cryptocurrency infrastructures ( Habib *et al.*, 2022). Opposite to cryptocurrencies however, where there is no central supervising authority, it is expected that central banks will issue their CBDCs in private blockchains, thus retaining the control of the chain ( PWC, 2019). Already, bank officials from the central banks of the Bahamas and the Caribbean are expressing great satisfaction about the security that blockchain offers ( Soderberg *et al.*, 2022). Another blockchain property that will prove integral for CBDCs is the capacity to create smart contracts, which can be employed to facilitate the automatic execution of the terms of an agreement and the carrying out of relevant transactions, without human intervention ( Bank of England, 2020). There are several publications that showcase the value of smart contracts for CBDCs. Indicatively, leveraged blockchain and smart contracts to ensure the automatic interoperability between CBDCs of different countries and the stability of said transactions. In another publication, the author proposed a CBDC architecture consisting of two layers, in which the distribution layer is built on a permissioned blockchain, and smart contracts are employed for various tasks and transactions ( Kumar, 2021). Moreover, ( Dash *et al.*, 2022) discussed the potential of a digital currency in India and emphasised the value of smart contracts in pre-defining conditions, agreements, and contracts between banking customers. Finally, ( Frankó *et al.*, 2022) presented a very interesting proposition of leveraging CBDCs to facilitate the transactions and interactions of industrial stakeholders with the banking system. In this case, smart contracts are envisioned to be used to automate and control the interactions between parties.

## CBDCs in the EU and legal framework

Across the European Union (EU), various efforts are underway to explore the creation of a digital euro, largely driven by the needs of industrial stakeholders seeking more efficient payment mechanisms ( Klein *et al.*, 2020). One of the main considerations is whether this digital euro will be implemented as a CBDC. If so, it could offer a robust infrastructure—comparable to the Single Euro Payment Area (SEPA)—to streamline transactions not only for interbank operations but also for retail and online payments ( Burkhard, 2022). The introduction of a European digital currency could also accelerate the shift from cash-based to secure digital transactions, with the central bank ensuring the security of payments ( Mooij, 2021). The European Central Bank (ECB) has been actively assessing the technical and regulatory conditions required for such a currency. A well-designed digital euro is seen as a potential tool for advancing the EU’s financial goals by offering a reliable and fast digital payment method, serving as a viable alternative to services currently dominated by non-EU providers. However, concerns have been raised about possible adverse effects: if not carefully designed, the digital euro might undermine financial stability or weaken monetary policy transmission by reducing reliance on commercial bank deposits ( ECB, 2020).

Predicting the impact of a digital euro in the EU poses significant challenges due to the uncertainty surrounding its demand from EU citizens, its design characteristics (which remain largely undecided), and the political, social, economic, and technological conditions that will be present in the EU society. This uncertainty is magnified by the fact that there have been no real-life applications of CBDCs by any country of the EU or even by advanced economical nations outside of the EU, which makes data availability scares at best.

Despite this uncertainty, the ECB seems to still be interested in exploring pathways to leverage this new monetary technology, as in a 2022 report, they set out the main goals and the application cases of a digital euro as well as matters that need to be examined such as the online and offline availability of the digital currency, the development of tools that would allow control over the amount of digital euros that are being issued and circulated, and issues of user privacy ( ECB, 2022).

When it comes to user privacy and the safeguarding of personal data, two main approaches are currently under consideration. One option involves categorising transactions based on their value and associated risk level to manage privacy and security more effectively. In this framework, which is comparable to existing digital banking practices, users would complete an initial identification process but could retain greater privacy for low-risk, low-value transactions. Conversely, high-value or high-risk payments would be subject to increased transparency and traceability, leveraging the technological features of CBDCs to ensure accountability among all parties involved ( Hall, 2022).

Another possibility under consideration is the implementation of an **"**offline mode" for CBDCs, which could enhance data privacy for smaller transactions, similar to the anonymity provided by cash payments. In this scenario, once users complete the initial identification and onboarding process, they will retain exclusive access to their account credentials. As a result, low-value transactions could be conducted without continuous online connectivity, while still preserving user privacy. In addition, a CBDC offline mode would be beneficial to the people who live in remote areas without access to smart devices and fast internet connections, which is a disadvantage of online payment systems in general ( ECB, 2022). However, the fact that as of the time of writing this publication, there are no immediate plans from the EU to publish and adopt a digital euro, in the form of a CBDC, gives European economists and policy makers ample time to tackle and set out the rules that will govern any regulatory, security, and other aspects of this new digital currency ( Handagama, 2022).

## CBDC potential for the green economy

Despite growing awareness of the need to investigate the environmental and developmental impacts of financial and monetary policies around the world, there is little relevant data on CBDCs. CBDCs are expected to bring about fundamental changes in monetary policy instruments, and therefore most of the related research has focused on their potential impact on financial stability and payment systems. However, research focusing on the environmental aspects of CBDCs and their contribution to sustainable development and the green economy is still at an early stage, which is to be expected considering that they are an unprecedented development, while countries that have fully implemented them are few. In the event of widespread adoption of these digital currencies, these issues will assume enormous importance as they directly relate to the long-term viability of an economy ( Lee & Park, 2022). After all, climate change affects, and is expected to affect increasingly in the future, monetary and financial stability through natural risks (such as extreme weather events and long-term climate changes), but also transition risks (i.e. behavioural changes, related to the demand for a greener world) ( Financial Stability Board, 2020). The overall environmental impact will not be limited to the loss of human life but will affect the overall financial stability and monetary policy tools available to a country ( Lee & Park, 2022).

An innovative application of CBDCs could be to target specific private "green" investments, reducing financing costs and providing valuable support to green business activities. In this way, they could be used as a vehicle to boost investments in specific areas of interest ( Dziwok & Jäger, 2021). Given that CBDCs have many inane characteristics that make them ideal for fast, easy, and secure payments ( Shkliar, 2020), they could be used to facilitate investments and donations to “green” projects without the need to involve intermediaries, the presence of which, increases transaction cost. In that way, investors would ensure that the entirety of their donation would feed into the green economy ( Ozili, 2022a). By eliminating investment fees and transaction costs (which are much higher for large-value transactions), the amount of funds that are invested in the green economy would rise significantly giving a much-needed boost to the domain ( Dziwok & Jäger, 2021).

Furthermore, it has been observed that it is a common phenomenon for workers, who are employed in businesses mainly in the circular economy (e.g. waste collectors) to be paid in physical currency, most likely because they do now have a personal bank account. This phenomenon is most common in developing and low-income countries and prohibits informal workers from being a part of the national banking system ( Wiener *et al.*, 2019). If CBDCs were adopted in these countries alongside the creation of a national digital wallet system, it would enable employers to compensate informal workers using digital currency—even in cases where the recipients do not hold a traditional bank account. This shift could offer several benefits: it would provide a secure alternative to cash, which is more vulnerable to loss, theft, or other risks, and it would grant these workers access to the formal financial system. With such access, they could begin to utilise banking services, including the ability to apply for loans and other financial tools ( Ozili, 2022b).

In addition, CBDC can be used as a means of offering financial assistance to businesses operating or investing in the Green Economy, and which face financial problems ( Riccardi & Levi, 2018). The use of CBDCs can create a direct link between businesses and the Central Bank, facilitating direct lending and avoiding traditional credit institutions and unnecessary bureaucracy. CBDCs could offer businesses a direct channel to request financial support from the Central Bank, along with more favorable conditions—such as reduced interest rates—when applying for loans. While this may not be a definitive justification for implementing CBDCs, it holds particular significance for green and circular economy enterprises. These types of businesses often aim to maximise their environmental impact rather than profit, which can limit their eligibility for conventional loans or make high-interest repayments unsustainable. In such cases, a Central Bank operating with CBDCs could digitally disburse loans under more accommodating terms, especially in situations requiring targeted financial intervention ( Ozili, 2022a).

CBDCs can also be used to provide immediate support to businesses in times of crisis, which would save them from a major loss of revenue or income ( Giudice *et al.*, 2020). Such crises can create liquidity problems, preventing businesses from paying workers' wages and repaying other obligations. With CBDCs, the government through the central bank can step in and provide funding to green economy businesses hit by a crisis (e.g., economic crisis, pandemic, natural disasters, war, etc.) by transferring the aid directly to the CBDC account ( Giudice *et al.*, 2020; Ozili, 2022a).

The relationship between CBDCs and the green economy is largely influenced by how the digital currency is designed, the specific goals set by the issuing Central Bank, and the extent to which it shapes financial activities related to environmentally sustainable initiatives. For example, a CBDC can be designed to offer transaction cost relief in order to incentivise waste reduction or other green and circular activities. It could also be used to give incentives for tax exemptions or tax discounts to companies active in the green economy. In this way, more and more businesses will be encouraged to use CBDC, leading to the faster growth of this industry. In addition, it could be designed in such a way as to limit its use to transactions by companies operating in the linear economy, by imposing higher taxes and commissions on transactions, thus further promoting the EU’s sustainable, green goals.

In addition to the above, businesses could also offer incentives to individuals to encourage environmentally responsible behaviors, facilitated through the use of CBDCs. One possible approach might involve setting energy reduction targets within electricity supply contracts and linking the amount of energy saved to a corresponding value in “green” digital euros. This would create a measurable and rewarding connection between reduced consumption and financial benefit. Citizens could be given the option to use earned green CBDCs as discounts on future electricity bills, serving as a financial reward for reduced energy consumption. Such mechanisms could be embedded directly into electricity contracts and executed automatically via well-structured smart contracts, thereby minimising administrative burdens for utility providers. Alternatively, these digital funds could be applied toward energy-efficiency upgrades in homes—aligning with the goals of the European Union’s "Renovation Wave for Europe" initiative (par. 4.3.3.1). This concept could also extend to other types of green investments, such as the installation of photovoltaic systems, with participating businesses offering additional incentives for CBDC users. Implementing these measures would require the establishment of a coordinated ecosystem involving public authorities, financial institutions, and private sector actors, all under EU oversight. This integrated approach would not only support CBDC adoption in everyday life but also accelerate the broader shift toward a sustainable, green economy.

## Conclusions and next steps

In this body of work, we have shown that CBDCs have the potential to play a transformative role in building a more sustainable economic future, especially by supporting initiatives related to renewable energy and environmental sustainability. Through a thorough examination of its potential applications, it is evident that CBDCs can serve as a catalyst for positive environmental change and energy efficiency.

CBDC offers a digital infrastructure that can revolutionise financial transactions, promoting efficiency and transparency in resource allocation. Leveraging blockchain technology and smart contracts, CBDC systems can ensure greater accountability in directing funds towards green initiatives and renewable energy projects, thereby enhancing the effectiveness of sustainability efforts. Moreover, the issuance of CBDC provides central banks with a powerful tool to incentivise and channel investments towards sustainable development. CBDCs can serve as effective tools for promoting environmental sustainability by guiding monetary policy and enabling digital payment systems that support investments in renewable energy and green initiatives. These mechanisms can help accelerate the shift toward a low-carbon economy. Beyond improving financial inclusion and fostering innovation, CBDCs may also open green investment opportunities to underserved populations, particularly in developing regions. With a secure and user-friendly digital infrastructure, they can support microtransactions and peer-to-peer lending for environmentally focused projects, encouraging broader public engagement in the green economy. Additionally, CBDCs offer the potential to address environmental externalities and promote more sustainable consumption and investment behaviors.

However, the integration of CBDC into the existing regulatory framework necessitates careful consideration of legal, technical, and governance challenges. Policymakers must collaborate with industry stakeholders to establish robust regulatory frameworks that safeguard against potential risks such as cybersecurity threats, money laundering, and privacy concerns.

Looking ahead, future research should explore the macroeconomic implications of CBDC adoption, its effectiveness in achieving sustainable development goals, and interdisciplinary collaborations to harness its full potential. International cooperation is essential to ensure interoperability and standardisation of CBDC systems across jurisdictions, fostering trust and confidence in CBDC as a tool for driving sustainable economic growth. In conclusion, CBDC holds immense promise as a transformative force in advancing sustainability objectives and shaping a greener, more resilient economy. Realising this potential requires concerted efforts from policymakers, regulators, and industry stakeholders to address technical challenges, ensure ethical standards, and promote inclusive growth. As we navigate towards a more sustainable future, CBDC stands poised to unlock new opportunities for economic prosperity and environmental stewardship.

## Ethics and consent

Ethical approval and consent were not required

## Data Availability

No data are associated with this article.

## References

[ref-3] Arner DW Auer R Frost J : Stablecoins: risks, potential and regulation.2020. Reference Source

[ref-9] Atlantic Council: CBDC tracker. (Accessed April 10, 2024). Reference Source

[ref-17] Auer R Böhme R : The technology of retail central bank digital currency. *BIS Quarterly Review.* March,2020. Reference Source

[ref-1] Auer R Frost J Gambacorta L : Central bank digital currencies: motives, economic implications, and the research frontier. *Annu Rev Econom.* 2022;14:697–721. 10.1146/annurev-economics-051420-020324

[ref-20] Bank of England: Central bank digital currency: opportunities, challenges and design.Discussion Paper,2020. Reference Source

[ref-8] Bech ML Garratt R : Central bank cryptocurrencies. *BIS Quarterly Review.* 2017; [accessed April 10, 2024]. Reference Source

[ref-25] Burkhard B : The digital euro – an opportunity for Europe. Speech at Cash Con 2022. Leipzig, 07.09.2022,2022.

[ref-2] Choi S : What promotes smartphone-based mobile commerce? Mobile-specific and self-service characteristics. *Internet Res.* 2018;28(1):105–122. 10.1108/IntR-10-2016-0287

[ref-5] CPMI: Central bank digital currencies. Bank for International Settlements,2018; [accessed April 10, 2024]. Reference Source

[ref-22] Dash B Ansari MF Sharma P : Future ready banking with smart contracts-CBDC and impact on the Indian economy. *Int J Netw Secur Appl.* 2022;14(5):5. 10.5121/ijnsa.2022.14504

[ref-34] Dziwok E Jäger J : A classification of different approaches to green finance and green monetary policy. *Sustainability.* 2021;13(21): 11902. 10.3390/su132111902

[ref-27] ECB: Report on a digital euro. Frankfurt: European Central Bank,2020; [accessed April 10, 2024]. Reference Source

[ref-29] ECB: Progress on the investigation phase of a digital euro. Frankfurt am Main: European Central Bank,2022; [accessed April 10, 2024]. Reference Source

[ref-33] Financial Stability Board, FSB: The implications of climate change for financial stability. 2020. Reference Source

[ref-23] Frankó A Olah B Sass Z : Towards CBDC-supported smart contracts for industrial stakeholders. In: *2022 IEEE 5th International Conference on Industrial Cyber-Physical Systems (ICPS).*IEEE,2022. 10.1109/ICPS51978.2022.9816857

[ref-39] Giudice F Caferra R Morone P : COVID-19, the food system and the circular economy: challenges and opportunities. *Sustainability.* 2020;12(19): 7939. 10.3390/su12197939

[ref-11] Gnan E Masciandaro D : Do we need central bank digital currency. *Conference Proceedings.* 2018;2. Reference Source

[ref-18] Habib G Sharma S Ibrahim S : Blockchain technology: benefits, challenges, applications, and integration of blockchain technology with cloud computing. *Future Internet.* 2022;14(11):341. 10.3390/fi14110341

[ref-30] Hall I : ECB to begin compiling digital euro rulebook. Global Government Fintech, October 05,2022; [accessed April 10, 2024]. Reference Source

[ref-31] Handagama S : Europe’s central bank lays out tough requirements for the digital euro to succeed. Fortune, July 14,2022; [accessed April 10, 2024]. Reference Source

[ref-10] Huynh K Molnar J Shcherbakov O : Demand for payment services and consumer welfare: the introduction of a central bank digital currency. Staff Working Paper 2020–7. Bank of Canada,2020. Reference Source

[ref-24] Klein M Gross J Sandner P : The digital euro and the role of DLT for central bank digital currencies. *Frankfurt School Blockchain Center Working Paper.* 2020. Reference Source

[ref-12] Koumbarakis A Dobrauz-Saldapenna G : Central bank digital currency: benefits and drawbacks.2019; Available at SSRN 3429037. Reference Source

[ref-21] Kumar S : Permission blockchain network based central bank digital currency. In: *2021 IEEE 4th International Conference on Computing, Power and Communication Technologies (GUCON).*IEEE,2021. 10.1109/GUCON50781.2021.9574020

[ref-15] Lee DKC Yan L Wang Y : A global perspective on central bank digital currency. *China Economic J.* 2021;14(1):52–66. 10.1080/17538963.2020.1870279

[ref-32] Lee S Park J : Environmental implications of a Central Bank Digital Currency (CBDC).2022. Reference Source

[ref-6] Mancini-Griffoli T Peria MSM Agur I : Casting light on central bank digital currencies. *IMF staff discussion note.* 2018;18(8):1–39. 10.5089/9781484384572.006

[ref-7] Mersch Y : Digital base money: an assessment from the ECB’s perspective. Speech at the Farewell ceremony for Pentti Hakkarainen. Deputy Governor of Suomen Pankki–Finlands Bank, Helsinki, 162017. Reference Source

[ref-26] Mooij AAM : European central bank digital currency: the digital euro. What design of the digital euro is possible within the European Central Bank’s Legal Framework? 2021. Reference Source

[ref-16] Olson Ø : Central Bank Digital Currencies. *Norges Bank Papers.* 2018; (1):5–31. Reference Source

[ref-4] Ozili PK : Circular economy and central bank digital currency. *Circ Econ Sustain.* 2022a;2(4):1501–1516. 10.1007/s43615-022-00170-0 35434721 PMC8989098

[ref-37] Ozili PK : Can central bank digital currency increase financial inclusion? Arguments for and against. *Big data analytics in the insurance market.* Emerald Publishing Limited,2022b;241–249. 10.1108/978-1-80262-637-720221013

[ref-14] PWC: The rise of Central Bank Digital Currencies (CBDCs) what you need to know.2019; [accessed April 10, 2024]. Reference Source

[ref-38] Riccardi M Levi M : Cash, crime and anti-money laundering. *The Palgrave handbook of criminal and terrorism financing law.* 2018;135–163. 10.1007/978-3-319-64498-1_7

[ref-35] Shkliar AI : The phenomenon of Central Banks' Digital Currencies (CBDC): key attributes and implementation perspectives. *Ukrainian Society.* 2020;1(72):123–137. 10.15407/socium2020.01.123

[ref-19] Soderberg G Bechara M Bossu W : Behind the scenes of central bank digital currency: emerging trends, insights, and policy lessons.2022. Reference Source

[ref-13] Wadsworth A : The pros and cons of issuing a central bank digital currency. *Reserve Bank of New Zealand Bulletin.* 2018;81:1–21. Reference Source

[ref-36] Wiener M Gall M de Oliveira CC : Circular business model innovation–insights from Mr. Green Africa.2019. Reference Source

