# Supplementary material for: An overview of CBDCs and their potential role in the green economy
Source: Open Res Eur. 2025 Apr 24;5:113. [Version 1] doi: 10.12688/openreseurope.19970.1 (PMC12181764; doi:10.12688/openreseurope.19970.1)
Supplement: Peer review history

# Reviewer report: Reviewer response for version 1

**DOI:** 10.21956/openreseurope.21601.r54372

**Published:** 2025-06-20

**Authors:** Sangita Gazi

**Affiliations:** 1 The Wharton School, University of Pennsylvania, Philadelphia, Pennsylvania, USA

The article provides a well-structured and informative overview of Central Bank Digital Currencies (CBDCs), covering their definition, taxonomy, global developments, advantages, disadvantages, technological background, and potential impact in the European Union (EU). It successfully introduces complex concepts in an accessible manner. However, the article's justification for how CBDCs, operating on blockchain or DLT, specifically *help* the green economy feels somewhat underdeveloped. While it lists several potential applications, it doesn't deeply address the inherent energy consumption concerns often associated with the underlying DLT (blockchain) itself, especially when discussing "green" initiatives. The link between the *technology* and the *environmental benefit* could be more robustly argued or qualified.

**Strengths:**

 - Identifies the research gap regarding the environmental impact of CBDCs

 - Proposes several plausible mechanisms for CBDCs to support the green economy, for example:

- Targeting green investments by reducing financing costs and eliminating intermediaries/transaction fees;
- Facilitating payments for informal workers in the circular economy, bringing them into the formal financial system.
- Offering financial assistance to green businesses with favorable terms and direct lending from the Central Bank.
- Incentivizing green behaviors through design features (transaction cost relief, tax exemptions, higher taxes on linear economy transactions)

**Areas for Improvement**

**The "How" of Green:** While the *applications* are listed, the article doesn't sufficiently explain *how* CBDCs, specifically due to their DLT/blockchain nature, are *better* suited for these green economy applications than existing digital payment methods. The advantages listed (fast, easy, secure, transparent, smart contracts) are generic benefits of digital payments and DLT. The core issue remains: if DLT has an energy cost, how does the *specific architecture* of a CBDC mitigate this, or how do the *benefits* outweigh that cost in the context of "green"?

**Missing Nuance on DLT's Environmental Footprint:** The article largely sidesteps the environmental impact of DLT itself. For a paper focusing on the "green economy," a critical discussion of the *energy efficiency* of permissioned DLTs (which CBDCs would likely use) versus public blockchains, and how this compares to traditional financial infrastructure, is essential. Without this, the "green" argument feels incomplete. It's not enough to say that blockchain offers security and transparency; it needs to explicitly state why this particular digital infrastructure is *environmentally beneficial* or *less harmful* than alternatives, or how its unique features enable *more effective* green initiatives that justify its own footprint.

**Lack of Specificity on "Green" Design:** While "designing a CBDC to offer transaction cost relief to incentivize waste reduction" is mentioned, more concrete examples or architectural considerations for this would strengthen the argument. How would a smart contract specifically implement a "green" discount or tax efficiently and transparently?

**Suggested approach**

 The authors may want to examine how technology, in general, supports green finance. There is literature that argues digital technologies produce reduced carbon footprints. The designs of CBDCs must consider the energy consumption implications of blockchain technology. Literature suggests that certain blockchain architectures are more environmentally friendly than others. Some references are suggested below. Yang Q et al 2023;  Akomea-Frimpong I et al 2022; Sandri D et al 2022; Shen Y et al 2023; Alzoubi Y et al 2023 (Ref 1 to 5)

Is the review written in accessible language?

Yes

Are all factual statements correct and adequately supported by citations?

Yes

Are the conclusions drawn appropriate in the context of the current research literature?

Partly

Is the topic of the review discussed comprehensively in the context of the current literature?

Partly

Reviewer Expertise:

My PhD research focuses on the legal and macroeconomic implications of central bank digital currencies.

I confirm that I have read this submission and believe that I have an appropriate level of expertise to confirm that it is of an acceptable scientific standard, however I have significant reservations, as outlined above.

## References

[1] : The Role of CBDC in Green Finance and Sustainable Development. *Emerging Markets Finance and Trade* .2023;59(15) : 10.1080/1540496X.2023.2185096 4158-4173 10.1080/1540496X.2023.2185096

[2] : A review of studies on green finance of banks, research gaps and future directions. *Journal of Sustainable Finance & Investment* .2022;12(4) : 10.1080/20430795.2020.1870202 1241-1264 10.1080/20430795.2020.1870202

[3] : Digital Currencies and Energy Consumption. *FinTech Notes* .2022;2022(006) : 10.5089/9798400208249.063 10.5089/9798400208249.063

[4] : Impact of digital technology on carbon emissions: Evidence from Chinese cities. *Frontiers in Ecology and Evolution* .2023;11: 10.3389/fevo.2023.1166376 10.3389/fevo.2023.1166376

[5] : Green blockchain – A move towards sustainability. *Journal of Cleaner Production* .2023;430: 10.1016/j.jclepro.2023.139541 10.1016/j.jclepro.2023.139541

# Reviewer report: Reviewer response for version 1

**DOI:** 10.21956/openreseurope.21601.r54050

**Published:** 2025-06-12

**Authors:** Silvana Prodan

**Affiliations:** 1 Babeș-Bolyai University, Cluj-Napoca, Romania

This study explores central banks' motivations for implementing Central Bank Digital Currencies (CBDCs) in the economy, focusing on financial resources, incentives for green investments, and their application as transactional or value-storage tools, specifically mentioning the European Central Bank's digital euro initiatives.

 The article has been well researched, is concise and covers the main aspects of CBDCs. Especially interesting is the chapter on CBDCs in the EU.

 No points to be addressed have been identified.

Is the review written in accessible language?

Yes

Are all factual statements correct and adequately supported by citations?

Yes

Are the conclusions drawn appropriate in the context of the current research literature?

Yes

Is the topic of the review discussed comprehensively in the context of the current literature?

Yes

Reviewer Expertise:

CBDCs

I confirm that I have read this submission and believe that I have an appropriate level of expertise to confirm that it is of an acceptable scientific standard.

# Reviewer report: Reviewer response for version 1

**DOI:** 10.21956/openreseurope.21601.r54370

**Published:** 2025-06-02

**Authors:** SERGIO LUIS NÁÑEZ ALONSO

**Affiliations:** 1 Catholic University of Ávila, Ávila, Spain

 The article “An overview of CBDCs and their potential role in the green economy” (provides a narrative review of the reasons why central banks are considering or issuing CBDCs and, more innovatively, how these digital currencies could support the transition to a green economy.

 Although the article presents an interesting take on a hot topic, in its current form it should not be accepted for indexing for a number of reasons, which I will outline below:

 1. Linking CBDCs to climate goals is timely, but the paper sticks to conceptual arguments; it doesn't give any empirical evidence or modeling.

 2. Source: Scopus, 2017–2022. Table 1 shows 8 topic searches with article counts. Lack of systematic rigor: complete search strings, inclusion/exclusion criteria, PRISMA protocol, and study quality assessment are not detailed. The time coverage is outdated: literature from 2023-2025 is excluded. Why do they only use Scopus and not WOS or other platforms such as OpenAlex with many more references?

 3. They explain well the token/account dichotomy and the difference between retail/wholesale. However, stating that wholesale CBDCs are “exclusively” token-based ignores account-based designs (e.g., the Helvetia project). Furthermore, it is argued that privacy “may be similar to cash” in offline mode, but the challenges for offline privacy are not discussed. In addition to the above, it is completely overlooked that several CBDC proposals do not use blockchain but centralized databases (e-Krona, FedNow Pilot).

 4. It uses the Atlantic Council CBDC Tracker for country counts. This tool is not up to date. I recommend using https://cbdctracker.org/, which is maintained, updated, and supported by leading CBDC entities such as Digital Euro Assoc., authors such as John Kiff, and is licensed by MIT. It should be added that, based on the use of the previous tool, the number of countries “3 launched, 36 pilot...” is out of date in April 2025 (Jamaica and Nigeria were added in 2024; DCash was relaunched). It is stated that “19 of the G20 are at an advanced stage” without citing a source or date for this statement.

 5. It presents potential examples: micro-incentives, direct loans, “green euros” linked to energy savings, but fails to discuss the risks of greenwashing. It does not assess the energy cost of the DLT infrastructure itself, which is critical in a “green” paper. It summarizes the ECB reports for 2020-22 and the debates on privacy/transaction types. However, the statement that “there are no live applications in advanced economies” ignores Hong Kong (mBridge), Singapore (Ubin), and Korea (wholesale pilot).

 6. Minor drafting issues. Incorrect use of terms: “inane ability/characteristics” should be innate. Several words split into syllables.

 7. Key 2023-25 papers (BIS Project Aurora, IMF Fintech Note 2024) are not cited, nor are many references indexed in 2023 and 2024.

 The coevolution effect of central bank digital currency and green bonds on the net-zero economy (2024) Energy Economics, 134, art. no. 107587 DOI: 10.1016/j.eneco.2024.107587

 Can Central Bank Digital Currencies be green and sustainable? (2023) Green Finance, 5 (4), pp. 603 – 623 DOI: 10.3934/GF.2023023

 The Role of CBDC in Green Finance and Sustainable Development (2023) Emerging Markets Finance and Trade, 59 (15), pp. 4158 – 4173 DOI: 10.1080/1540496X.2023.2185096

 Sustainable Development in the Digital Age: The Role of CBDCs in Achieving the SDGs (2024) Contributions to Finance and Accounting, Part F4082, pp. 491 – 502 DOI: 10.1007/978-3-031-75960-4_45

 DIGITAL CURRENCY OF THE CENTRAL BANKS: TRENDS OF THE EURO AREA AND PROSPECTS OF THE USE WITHIN THE IMPLEMENTATION OF THE EUROPEAN GREEN DEAL (2024) Journal of Theoretical and Applied Information Technology, 102 (7), pp. 2954 - 2967

 8. Several URLs lead to generic or broken pages (check Bank of England 2020).

Is the review written in accessible language?

Yes

Are all factual statements correct and adequately supported by citations?

Partly

Are the conclusions drawn appropriate in the context of the current research literature?

Partly

Is the topic of the review discussed comprehensively in the context of the current literature?

Partly

Reviewer Expertise:

CBDC

I confirm that I have read this submission and believe that I have an appropriate level of expertise to confirm that it is of an acceptable scientific standard, however I have significant reservations, as outlined above.
